# Lessons learned from the achievement and implementation of interprofessional education in Thailand

**DOI:** 10.5116/ijme.60b7.6ec6

**Published:** 2021-06-25

**Authors:** Chanuttha Ploylearmsang, Sirinart Tongsiri, Methee Piriyakarnnon, Tharinee Srisaknok

**Affiliations:** 1 Social Pharmacy Research Unit, Faculty of Pharmacy, Mahasarakham University, Thailand; 2 Faculty of Medicine, Mahasarakham University, Thailand; 3 Faculty of Architecure, Urban design and Creative Arts, Mahasarakham University, Thailand

**Keywords:** Lessons learned, interprofessional education, Thailand

## To the Editor

Interprofessional education (IPE) was originally implemented by three faculties: Medicine, Pharmacy, and Architecture, Mahasarakham University (MSU), in Thailand. The highlights of this multidisciplinary IPE were that students in health-related and non-health-related disciplines blended their different ideas and attitudes in order to deliver a holistic care regime for patients who received care in their homes. It was provided through the three curricular (Intra-curricular activities) of the three faculties. It is hoped that it will result in the provision of humanized home-based care that is delivered by a group of students, with a teacher as the facilitator. For the first two years of the program in Thailand, it was envisaged that processes might be discovered that ensure that IPE can be implemented successfully and sustainably in terms of home-based care.

Home-based care was used as a learning process.^1^ Thirty patients in a selected area were visited at their homes by thirty groups of students. Three concepts: family medicine (INHOMESSS^2^, holistic approach of data gathering from patients, which includes the concepts of Immobility, Nutrition, Housing, Other people or family genograms, Medication, Physical Examination, Spiritual Health, Safety, Services), drug use and storage in a house, and Universal Design for each patient, were employed. Three types of preparation were carried out by an IPE working group that consisted of educators, students, and the community. Educators provided an inter-professional learning atmosphere in which students could form an inter-professional team.^2^ For IPE students, ice-breaking activities were carried out in order to help with team building and to make students from different faculties blend into a single unit. The planned learning outcomes for IPE students were created and evaluated. For patients, the relevant results were their satisfaction with the students' performance.

We have learned that six steps were involved in the achievement of relevant outcomes by students and patients: Step 1: Understanding their institutional philosophy and values. Teachers' attitudes towards university and IPE is the most important part of being an agent of change; Step 2: demonstrating shared experience and building understanding of the IPE concept and willingly integrating it into curricular; Step 3: identification of those who can provide leadership. Initiators who have a vision of interprofessional practice (IPP) for patient safety, are key agents of interprofessional education advocacy; Step 4: setting up an IPE working group. It should not be too small or too big, must be clear in its IPE principles (small and sharp), and needs to be able to spend informal time creating a positive atmosphere for creative work; Step 5: plan and prepare well in terms of three components. Teacher preparation: learning the concepts and principles of IPE, the academic year of the student, related courses and grading, learning outcomes, methods of measurement, and joint tool development^3^; Student preparation: providing them with basic knowledge and a platform for working as a team in order to plan their work together^4^; Community preparation: ensuring the readiness of patients in the community for home visits. PCU officers and health volunteers were prepared to be involved in the provision of teaching, evaluation, and creative innovation which were designed by groups of students;  Step 6:  continuing to learn and share by using After Action Reviews (AAR), in which the effectiveness of IPE will be discussed.

In summary, home-based inter-professional education was originally implemented in three faculties of Mahasarakham University, Thailand, in 2015.^5^ Three primary health care concepts were used. The main outcomes were improved attitudes towards interprofessional teamwork on the part of students from different disciplines and patients' satisfaction with the performance of students with regard to home care. From the lessons that were learned about the achievement of relevant outcomes, a six-step process of IPE implementation was created and utilized. The six steps are 1) understanding of institutional philosophy and values; 2) demonstrating shared experience and building understanding of the IPE concept; 3) identifying natural leaders; 4) setting up an IPE working group; 5) planning and preparing teachers, students, and communities;  6) continuing to learn and share together. This can be useful for educators when setting up IPE curricular in their institutes.

### Conflict of Interest

The authors declare that they have no conflict of interest.
